# *Histochemistry and Cell Biology*—a glance into the past and a look ahead

**DOI:** 10.1007/s00418-023-02195-4

**Published:** 2023-05-17

**Authors:** Jürgen Roth, Douglas J. Taatjes

**Affiliations:** 1 University of Zurich, CH-8091 Zurich, Switzerland; 2 Department of Pathology and Laboratory Medicine, Larner College of Medicine, University of Vermont, Burlington, VT 05405 USA

**Keywords:** Enzyme histochemistry, Carbohydrate histochemistry, Protein histochemistry, Light microscopy, Electron microscopy, Spectroscopy, MALDI-IMS

## Abstract

At the occasion of the 65th anniversary of *Histochemistry and Cell Biology*, we browse through its first ten years of publication and highlight a selection of papers from the early days of enzyme, protein, and carbohydrate histochemistry. In addition, we narrate recent progress to identify, quantify, and precisely determine the tissue localization of proteins and lipids, and small molecules by the combination of spectroscopic techniques and histology.

“*For the histologist, every progress in staining technique comes to be something like the acquisition of a new sense open to the unknown*” – Santiago Ramon y Cajal

## Introduction

As is often typical for any new emerging scientific branch, the opportunity to publish histochemical papers were initially somewhat limited due to lack of specific journals. During the 1950s, several journals exclusively dedicated to the publication of histochemical and cytochemical studies were established, including the *Journal of Histochemistry and Cytochemistry* (1953), *Acta histochemica* (1954), *European Journal of Histochemistry* (1954), *Annales d’Histochimie* (1956), and *Acta Histochemica et Cytochemica* (1959). *Histochemistry and Cell Biology*, as the journal is called nowadays, was launched in 1958 as the Division of Histochemistry of the present-day journal *Cell and Tissue Research* (Roth et al. 2008). To commemorate the occasion of its 65th anniversary, we will take a look at the kind of work that was published during the early days of the journal *Histochemie* (nowadays *Histochemistry and Cell Biology*). Without doubt, these studies have provided many conceptual and methodical breakthroughs still echoed in our current work. In addition, we will highlight a selection of groundbreaking papers published in *Histochemistry and Cell Biology* over the years.

### The early days of the journal (1958–1968)

**Fig. 1:**
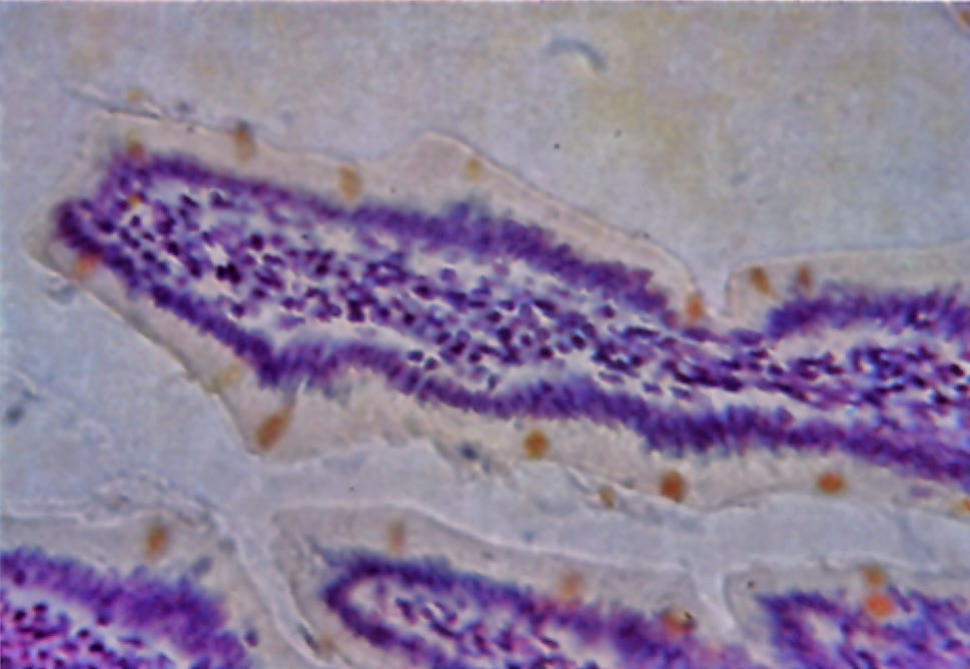
Section of mouse intestine stained with Feulgen reaction (cresyl violet-SO_2_) followed by PAS reaction (chrysoidine R-SO_2_). Formalin fixation and paraffin embedding. Figure 2 from Kasten 1959

Histochemical analysis of tissue and cell constituents during the 1950s and 1960s was certainly not an easy task. Unlike today, reagents such as antibodies, lectins, and staining kits were not commercially available and even self-made ones were rare. Moreover, to cite Michael Arnold (2007) “at this time there was a lot of skepticism among the leading morphologists (both anatomists and pathologists) with regard to histochemistry. The specificity of histochemical methods as well as the preservation of structures usually was rather poor.” Basically, the primary experimental choice available during this classical period was to apply specific chemical reactions on tissue sections, often with the use of particular dyes. The paper by Kasten (1959) detailing Schiff-type reagents, other than basic fuchsin, for the detection of nuclear and non-nuclear aldehyde groups ran to 43 pages in length and is a prime example of the arduous work involved. He investigated 435 batches of 216 different dyes, identifying 39 of them as aldehyde-specific reagents. The results of his experimental trials were illustrated with 19 figures, 12 tables, and abundant structural formulas. Lastly, he recommended 25 dyes for use in histochemistry, and demonstrated their specificity for aldehydes “by extensive use of aldehyde blocking reagents, treatment with formaldehyde, chemical extraction procedures, specific enzymatic tests, film tests, omission of essential aldehyde-producing steps for certain control sections, and identical optimal hydrolysis times in the Feulgen reaction.” Staining tissue sections with these Schiff-type reagents resulted in a yellow, orange, brown, red, violet, blue, or green coloration; notably, some of the dyes even produced a fluorescent staining signal. Therefore, double-aldehyde staining procedures to demonstrate aldehydes by the Feulgen reaction and the periodic acid–Schiff (PAS) reaction became possible (Fig. 1).

The paper by Gössner (1961) concerning the comparative analysis of amyloid, hyalin, and collagen is also noteworthy since it additionally offered a comprehensive appraisal of techniques to demonstrate specific amino acids and various protein groups, such as sulfhydryl and α-amino groups, at that time. The demonstration of protein carboxyl groups could be achieved by techniques using acetic anhydride-pyridine (Karnovsky and Mann 1961) or carbodiimide (Geyer and Schulz 1968).

**Fig. 2:**
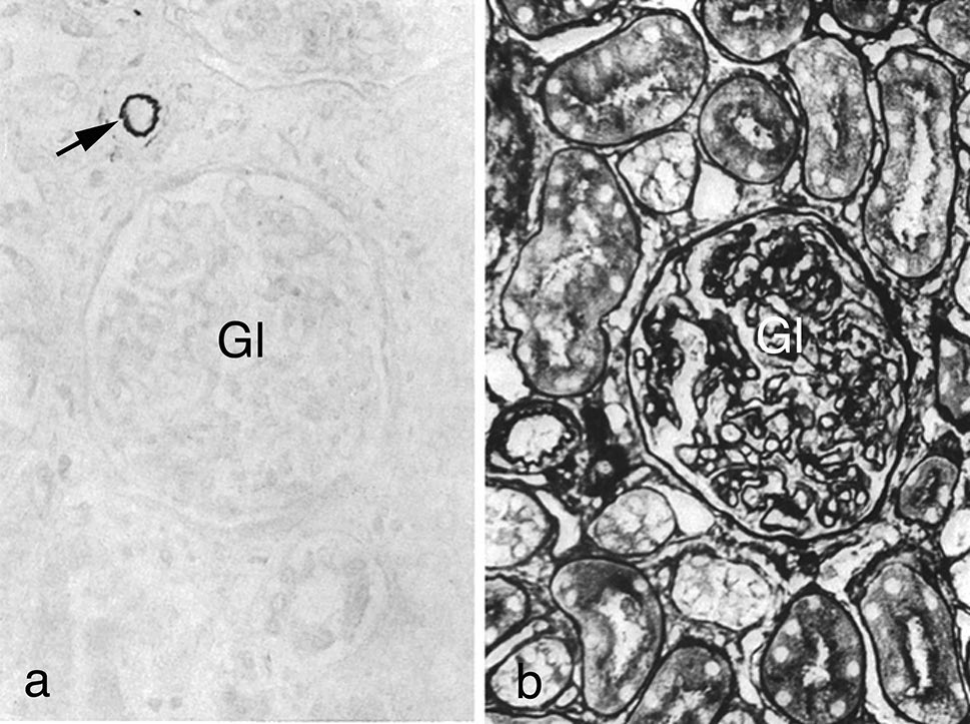
Human kidney sections. **a** Elastic fibers of a small artery (arrow) are stained with resorcin-fuchsin. Gl: glomerulum. **b** Sulfation prior to resorcin-fuchsin staining results in coloration of glomerular basement membranes, Bowman’s capsule and tubules, reticulum fibers, and cytoplasm. Fixation in absolute alcohol and embedding in paraffin. Figures 1 and 2 from Puchtler and Sweat 1964b

A striking example of the interconnection between chemistry and histology is the paper by Puchtler and Sweat (1964a) “On the mechanism of sequence iron-hematein stains” for the demonstration of iron bound to tissue structures. The iron alum-hematein stain exhibited some degree of specificity by staining the A and Z bands and nuclei of striated muscle fibers, intercalated discs of cardiac muscle fibers, smooth muscle fibers, fibrin, tonofibrils, and terminal bars. In contrast, collagen, elastic and reticulum fibers, and basement membranes did not display staining. Prior deamination of the tissue sections enhanced the staining, whereas methylation or acetylation abolished it. Thus, it was concluded that nitrogen atoms of amino groups were not electron donors in the binding of iron to tissues, but carboxyl and hydroxyl groups were indispensable. Fittingly, these findings were in good agreement with the results of chemical experiments performed in reagent tubes. In terms of histochemistry, the authors concluded that tissue structures belonging to the “keratin-myosin-epidermin-fibrinogen group”^1^ were stained by the iron alum-hematein, whereas those belonging to the “collagen group” were not. In another study by Puchtler and Sweat (1964b), the specificity of resorcin-fuchsin and van Gieson picro-fuchsin type stains, including 67 dyes both histological and textile, for connective tissue fibers was systematically evaluated and discussed in terms of the chemistry of the interaction between the dyes and tissue structures. In human kidney and rat liver sections, resorcin-fuchsin stained only elastic fibers (Fig. 2a). However, prior acetylation, sulfation, or phosphorylation of tissue sections resulted in additional staining of basement membranes, reticulum fibers, and collagen (Fig. 2b). Moreover, a selective staining of basement membranes and reticulum fibers by van Gieson type stains was observed. Taken together, although a certain relation between the tissue staining pattern and the class of protein(s) could be revealed, the limited degree of specificity achieved with these methods was typical for that time.

There were, however, some notable exceptions to this generalization. For instance, Schiebler and Schiesser (1959) succeeded in developing a method for the selective staining of insulin in pancreatic beta cells using pseudoisocyanine, in particular NN'-diethyl-6,6'-dichloropseudocyanine chloride. Importantly, prior oxidation of the tissue sections with an acidic KMnO_4_ solution was an obligatory step in the procedure. This technique resulted in a metachromatic staining of cytoplasmic granules in beta cells, whereas glucagon-producing alpha cells in the islets of Langerhans, exocrine pancreatic acinar cells, and mast cells were not stained. The histochemical results were validated by spectrophotometric analysis of isolated insulin, sequentially subjected to oxidation and incubation with pseudoisocyanine. On the basis of their experimental evidence, the authors concluded that (a) the highly specific metachromatic reaction for insulin was due to the formation of SO_3_^−^ groups resulting from the oxidation of its disulphide bonds and (b) required the presence of at least two very closely positioned SO_3_^−^ groups. The same principle was applied by Wolf (1965) to selectively stain the thyreotropin-secreting cells of the pituitary of humans and several animal species. In this protocol, as a first step, tissue sections were oxidized with performic acid, followed by incubation with dichloro pseudoisocyanine, which yielded a strong metachromatic reaction. This resulting metachromatic reaction was attributed to the presence of cysteine residues whose disulfide groups were oxidized to sulfo groups.

**Fig. 3:**
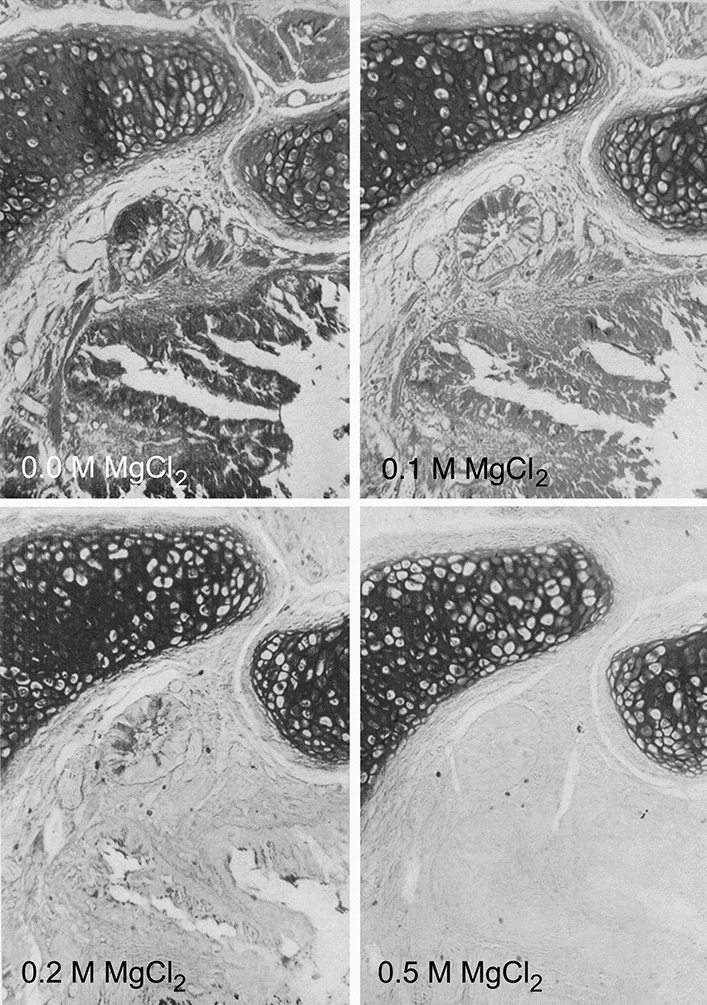
Consecutive sections from human newborn lung, alcian blue staining, and application of the critical electrolyte concentration. Increasing concentrations of magnesium chloride result in increasing selectivity of the staining. Formaldehyde fixation and paraffin embedding. Figures 5, 6, 7, 8 from Scott and Dörling 1965

**Fig. 4:**
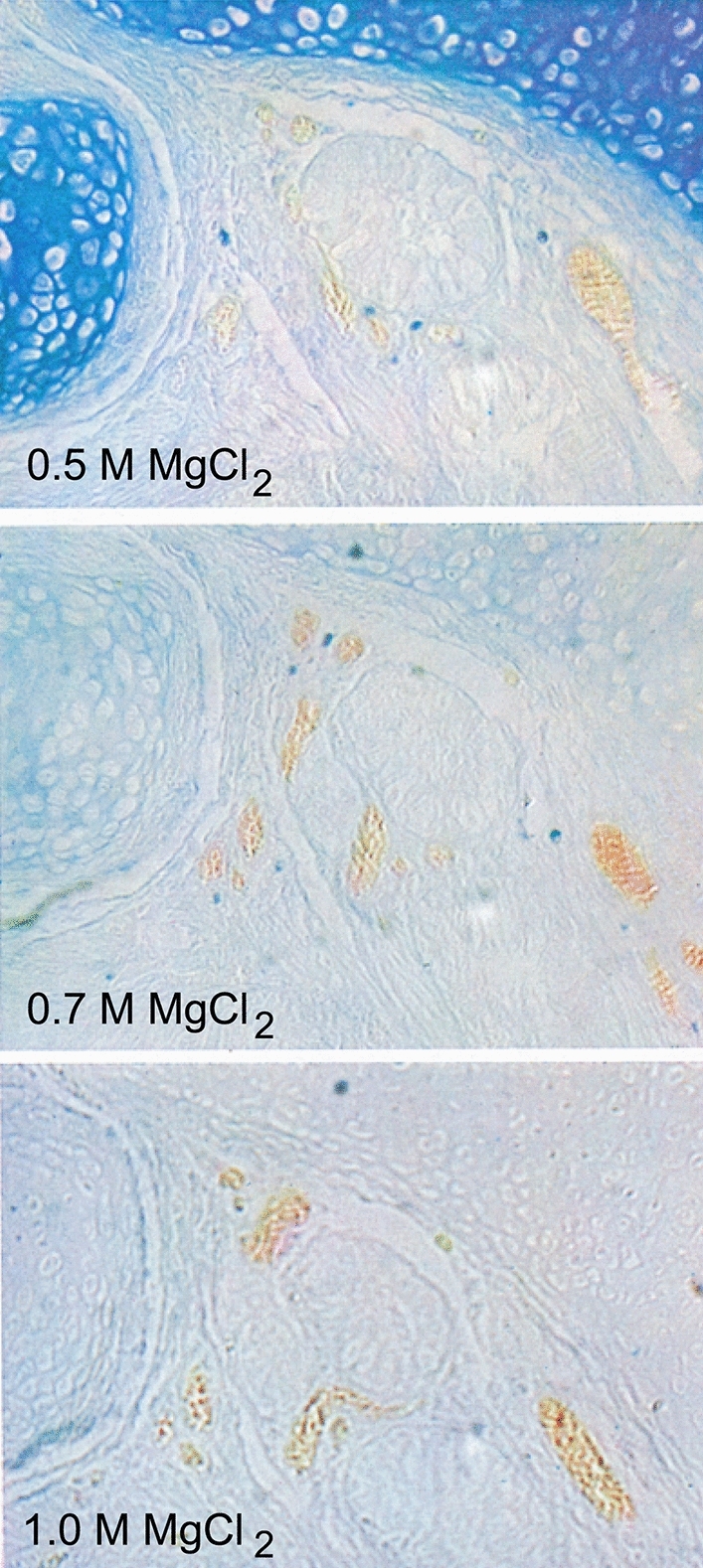
Consecutive sections from human newborn lung, alcian blue staining, and application of the critical electrolyte concentration. Addition of 1.0 M magnesium chloride to the alcian blue solution results in abolition of the cartilage staining. Formaldehyde fixation and paraffin embedding. Figures 9, 10, 11 from Scott and Dörling 1965

Investigators seeking at the detection of cellular carbohydrates had a rather limited repertoire of reagents from which to choose. Actually, at that time, the periodic acid–Schiff-based technique and the use of polycationic dyes (most often alcian blue 8GX) were not only quite popular, but had also been thoroughly explored chemically. Whereas the periodic acid–Schiff-based technique principally detected neutral polysaccharides, characterized by the presence of hexose and methylpentose residues containing free 1,2-glycol groups, the alcian blue method preferentially stained acidic (polyanionic) polysaccharides. In a series of seminal and beautifully illustrated papers, Scott and coworkers (Quintarelli et al. 1964a, b, 1965; Scott and Dorling 1965; Scott et al. 1964) investigated the mechanism of alcian blue staining, its binding to tissue polyanions, the impact of chemical blocking and unblocking of specific radicals, and the application of the critical electrolyte concept for staining and differentiation of acidic glycosaminoglycans (mucopolysaccharides). The application of the critical electrolyte solubility resulted in the discrimination of both carboxylated and sulfated polysaccharides in cartilage and various mucous-producing glands, while in addition, age-related and topographical differences in human costal cartilage could be revealed (Figs. 3 and 4). As pointed out by Mowry (1960), the contrasting coloration produced by the different reactions and dyes allowed the detection of chemically different mucins in tissue sections. Another enlightening paper in the same area was the study by Karnovsky and Mann from 1961. They used acetic anhydride-pyridine for the demonstration of carboxyl groups of proteins, as well as alcian blue, azure A, and aldehyde-fuchsin for acid mucopolysaccharides to investigate hyaline, elastic, and fibrous cartilage. Importantly, in these revelatory studies they also utilized isolated chondromucoprotein as a test substance for the staining reactions. They were able to demonstrate that chondromucoprotein of the cartilage matrix was detected with these methods and that differences in staining intensity “may in large part be related to the degree of aggregation of chondromucoprotein.” Of note, the authors concluded: “Observations made with this method on the staining of cartilage matrix in various physiological and pathological states would be of interest.”

**Fig. 5:**
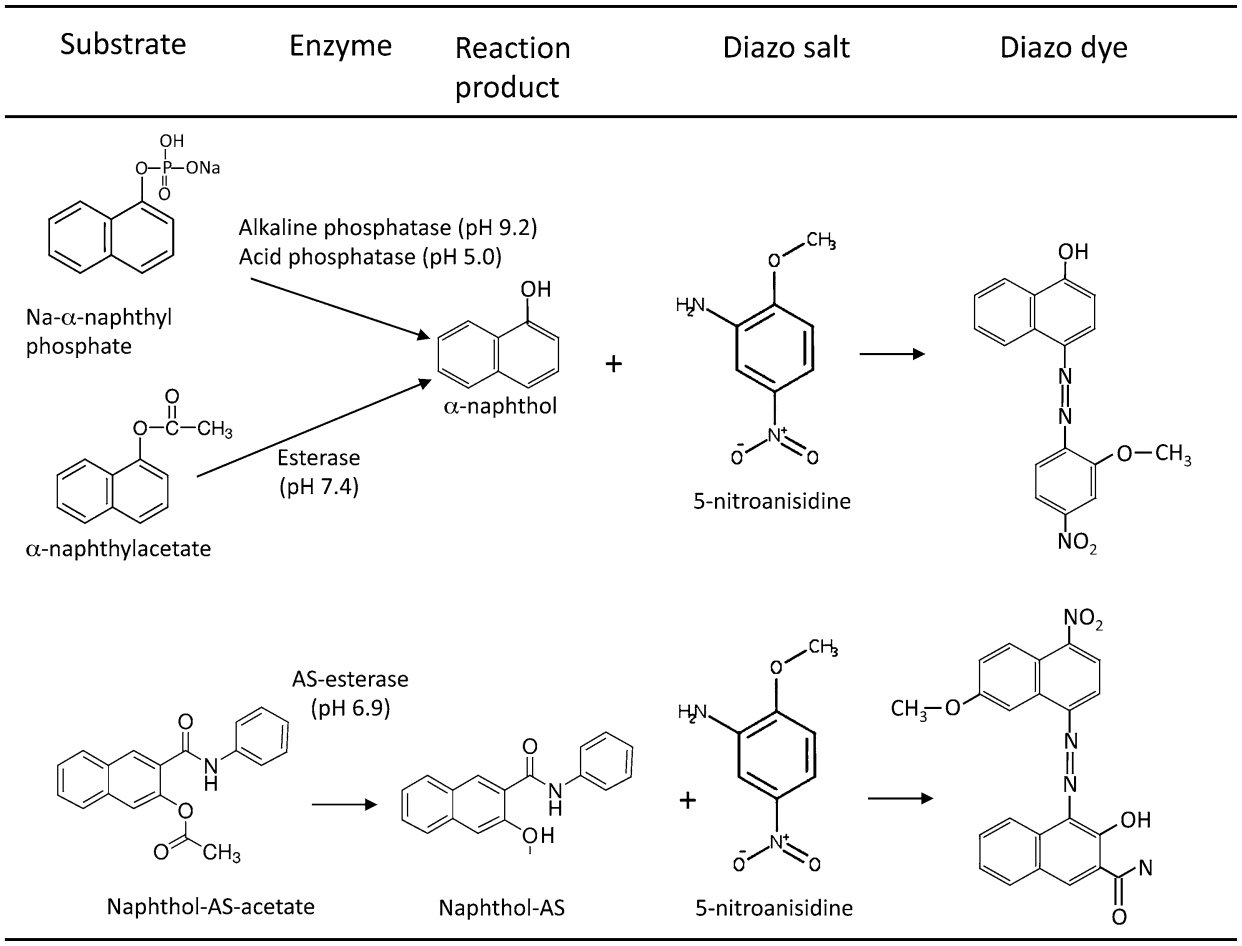
Schematic representation of enzyme histochemical reactions for phosphatases and esterase with the azo dye method. Adapted from Gössner 1958

**Fig. 6:**
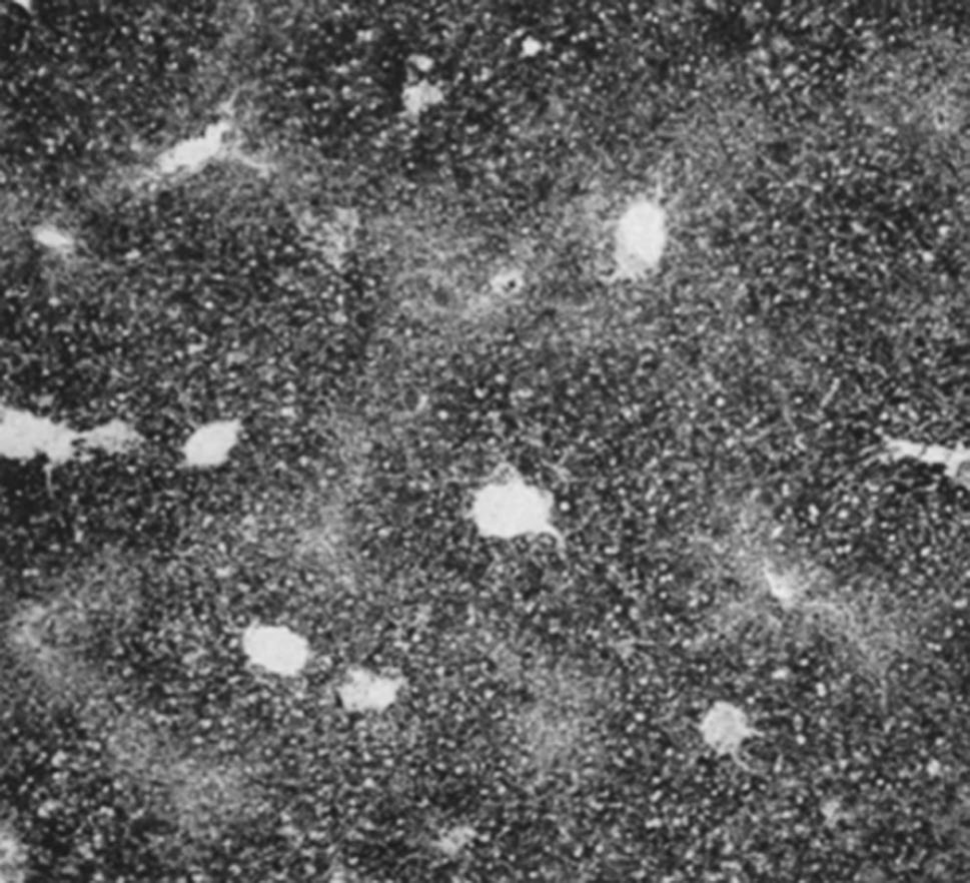
Demonstration of sulfatase activity in mouse liver with the azo dye method. Enzyme activity is detected in the hepatocyte cytoplasm and strongest in central region of the hepatic lobuli. Naphthol-AS-sulfate was used as enzyme substrate at pH 6.2 and Echtrot TR as diazo salt. Figure 3 from Gössner 1958

Another main area of investigation at that time was light and electron microscopic enzyme histochemistry by applying basic principles of enzymology to tissue samples. Essentially, the soluble reaction product resulting from the interaction between the enzyme activity of the tissue section and the added enzyme substrate was chemically coupled with a dye or precipitated with metal salts (Fig. 5). The location of the dye or the metal precipitate was interpreted as representing the in situ tissue distribution of the respective enzyme activity. General problems encountered in enzyme histochemistry were related to the preservation of enzyme activity by tissue fixation, the specificity of enzyme substrates, reaction product diffusion, non-specific precipitates, and structural preservation for electron microscopy. A most comprehensive treatise of the azo dye methods for the light microscopic demonstration of acid phosphatases and esterases was published by Gössner (1958). Using this method, he could demonstrate marked variation in the presence and distribution of the two enzyme families in kidney, liver, spleen, and pancreas (Fig. 6). Another exemplary work from this time was the paper by Eränkö (1959) describing acetylcholinesterase and non-specific cholinesterase in rat adrenal gland. He used acetylthiocholine and butyrylthiocholine as well as α-naphthyl acetate as substrates, and eserine (di-isopropylfluorophosphate), 62.C.47 (1:5-bis-(4-trimethylammoniumphenyl)-pentan-3-one di-iodine) and iso-OMPA (tetra-isopropylpyrophosphoamide) as enzyme inhibitors. In contrast to the specific demonstration of acetylcholinesterase using acetylthiocholine as substrate, butyryllthiocholine and α-naphthyl acetate revealed only activity for non-specific cholinesterase. This conclusion could be reached by the use of the different enzyme inhibitors and careful comparative analysis of the obtained tissue staining pattern. The pattern seen with butyryllthiocholine or α-naphthyl acetate were the same irrespective of the use of inhibitor 62.C.47. However, iso-OMPA completely abolished the reaction with these substrates. On the other hand, iso-OMPA completely abolished the reaction using acetylthiocholine, which could be completely inhibited using eserine. Thus, addition of iso-OMPA to the acetylthiocholine substrate enabled the specific demonstration of acetylcholinesterase, whereas addition of 62.C.47 to the butyryllthiocholine substrate specifically revealed non-specific cholinesterases. This observation was the basis for the successive demonstration of the two enzyme activities in the same tissue section. It should be noted that in the 1960s, the first studies with fluorescein isothiocyanate-labeled antibodies raised against enzymes were published. In continuation of earlier work on ribonuclease Ehinger and Lagerstedt (1959) and Ehinger (1965) prepared an antibody against ribonuclease, labeled it directly with fluorescein isothiocyanate, and detected the enzyme in the basal cytoplasm of acinar pancreatic cells by fluorescence microscopy. However, problems with regard to immunization protocols, antibody purity, and specificity, as well as non-specific binding, were encountered, while the available microscopic equipment was imperfect.

**Fig. 7:**
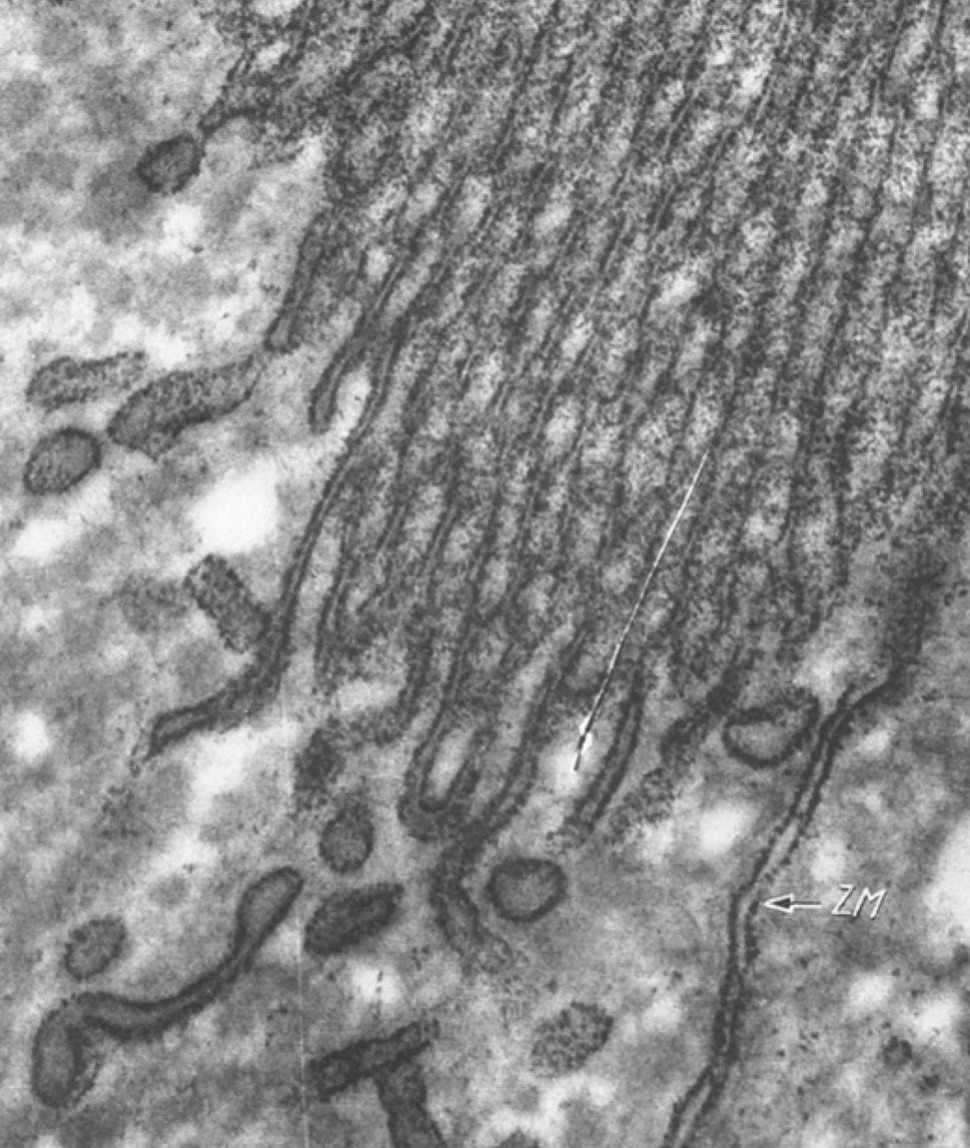
Electron microscopic demonstration of phosphatase in mouse kidney. An electron dense lead phosphate precipitate is present in the brush border of a proximal tubular epithelial cell and along the lateral plasma membrane. Figure 8 from Mölbert et al 1960

**Fig. 8:**
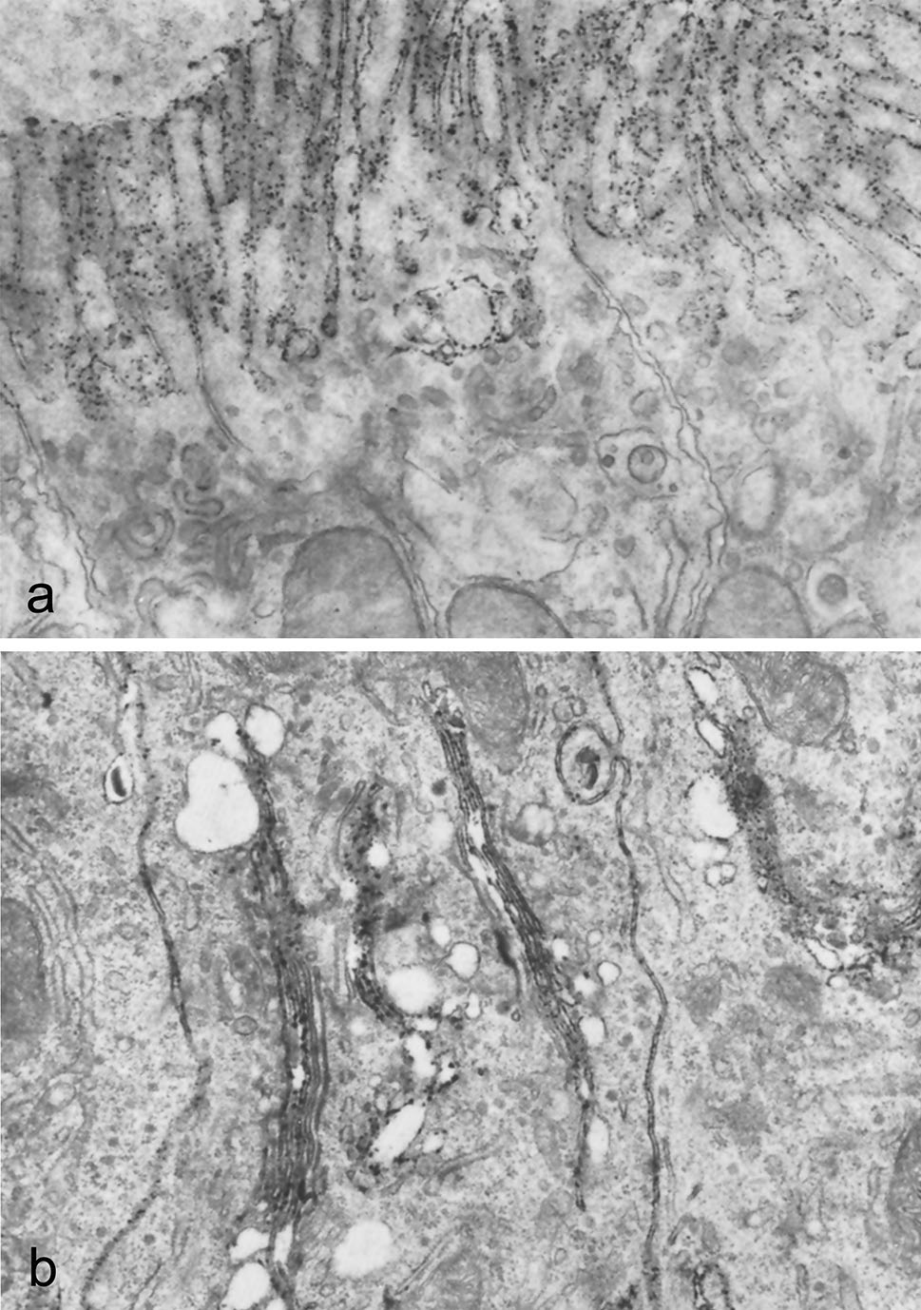
Electron microscopic demonstration of non-specific alkaline phosphatase. **a** In rat kidney, the electron dense, granular lead phosphate precipitate is evident in the brush border. **b** In the small intestine, the enzyme activity is detectable in the lateral plasma membrane and the Golgi apparatus, as well as the brush border (not shown). Figures 1 and 4 from Mayahara et al. 1967

Attempts to demonstrate enzymes by electron microscopy were hampered by diverse technical problems including unsatisfactory tissue structural preservation and limited enzyme specificity. In this regard, Mölbert et al. (1960) successfully modified the phosphatase technique of Gomori and Benditt (1953) by using lead nitrate as capture reagent in conjunction with brief osmium prefixation. In mouse kidney, a highly electron dense, fine lead phosphate precipitate was formed along the apical (brush border), lateral, and basal plasma membrane of well-preserved proximal tubular epithelia (Fig. 7). Moreover, Ericsson and Trump (1965) modified the Gomori technique (Gomori 1941) for acid phosphatase using formaldehyde and glutaraldehyde as fixative. In well preserved rat kidney and liver, a granular reaction product for acid phosphatase activity was exclusively formed in lysosomes, as could be anticipated by the biochemical work of de Duve and colleagues (Appelmans et al. 1955). Ogawa and coworkers (Mayahara et al. 1967) were similarly successful in the demonstration of activity for non-specific alkaline phosphatase using lead citrate as capture reagent at high alkaline pH in sections of aldehyde-fixed rat kidney, intestine, and cerebrum. Specific electron dense lead phosphate precipitate was observed in the plasma membrane and the Golgi apparatus (Fig. 8).

Coincident with these methodologically-driven studies, numerous functionally oriented analyses were performed as well. Daems and coworkers (1964) reported a method using 5,6-dihydroxy indoles and hydrogen peroxide for the detection of intravenously injected lactoperoxidase in rat liver by electron microscopy. In hepatocytes and Kupffer cells 1 hour after injection of the peroxidase, multiple pleomorphic membrane-limited structures in the cytoplasm between the Golgi apparatus and bile capillaries were stained. These structures were interpreted to represent lysosomes, although retrospectively many of these structures could have been endosomes. In hindsight of course, in 1964 the concept of endocytosis and the various endocytic structures were not known. The work by Pearse and coworkers was paradigmatic for the combination of methods and their application. Dubowitz and Pearse (1960) reported clear-cut evidence for the existence of two types of muscle fibers based on their reciprocal staining for oxidative enzymes and phosphorylase. The authors suggested that red (small) and white (large) skeletal muscle fibers “carry out their contractile functions by means of entirely different energy-supplying systems.” Thomas and Pearse (1961) detected regional and cell type-related distribution pattern of dehydrogenases in the central and peripheral nervous system. They ascertained good agreement of the histochemical findings with results from biochemical analysis and microassays. The painstaking studies by von Deimling and coworkers investigating “hormone dependent enzyme distribution in animal tissue” began with the analysis of alkaline phosphatase in the kidney of adult rats, revealing a specific male and female type of distribution and intensity (von Deimling and Noltenius 1964). After orchiectomy, no significant change in enzyme distribution occurred. However, subsequent estradiol treatment resulted in a switch of the male to the female enzyme staining pattern. In contrast, ovarectomy resulted in a strong reduction of enzyme activity without an additional effect of subsequent testosterone treatment (Baumann et al. 1964). Analogous studies and experiments in mice revealed sex-related differences for alkaline phosphatase and glucose-6-phosphatase activity (von Deimling et al. 1965). However, the effect of castration, testosterone, and estradiol treatment were different from those observed in rats. Further investigations analyzed the possible importance of the adrenal and pituitary gland on the renal alkaline phosphatase in normal, castrated, and hormone-treated rats (von Deimling et al. 1966), the effect of adrenalectomy and hypophysectomy (von Deimling et al. 1967), the actinomycin effect on estradiol-induced increase of alkaline phosphatase (von Deimling and Nemitz 1967) and the time-course of the estradiol-induced increase of renal alkaline phosphatase in orchiectomized rats. Moreover, enzyme histochemical studies were performed in diseased tissues as well. For instance, Allison and Burstone (1964) analyzed alkaline and acid phosphatase and esterase activity in mice infected with mouse hepatitis virus MHV3. They observed a progressive, generalized increase in peribiliary alkaline phosphatase, a localized increase in acid phosphatase and a decrease in esterase accompanying tissue damage and subsequent necrosis. Riecken and Pearse (1965) described alterations of acid phosphatase activity of the small intestine of patients with idiopathic steatorrhea before and during gluten-free diet. Hugon and Borgers (1966) analyzed the changes of acid phosphatase, thiamine pyrophosphatase, and ATPase in mice duodenal crypt stem cells accompanying fine structural alterations following whole-body supralethal X-ray irradiation.

Taken together, the works reviewed above that were published during the early days of this journal, and of its sister journals, are both remarkable and inspiring. They demonstrated that fundamental aspects of the chemical composition of tissues and cell types under physiological and pathological conditions could be disclosed with the ingenious use of chemistry-based methods.

Diverse other studies are noteworthy in addition to those highlighted above. Trace amounts of metals such as iron, tin, copper, cadmium, and lead could be detected in sections of kidney, preferentially in podocytes and proximal tubular cells, and liver with a silver sulfide reaction (Timm 1960; Timm and Neth 1959). De Vries and Meijer (1968) developed a histochemical method combined with a mineralization technique for the demonstration of titanium and iron oxides in pulmonary dust deposits. As pointed out by the authors, the technique was primarily developed to detect those oxides in the lung tissue of workers who had developed pneumoconiosis as an occupational disease. Histoautoradiography was applied for the demonstration of phosphorylases and glycogen synthetase (Guha and Wegman 1966, 1968). For the demonstration of the latter, the authors themselves (sic!) had to prepare ^14^C-glucose-6-phosphate enzymatically from ^14^C–glucose.

**Fig. 9:**
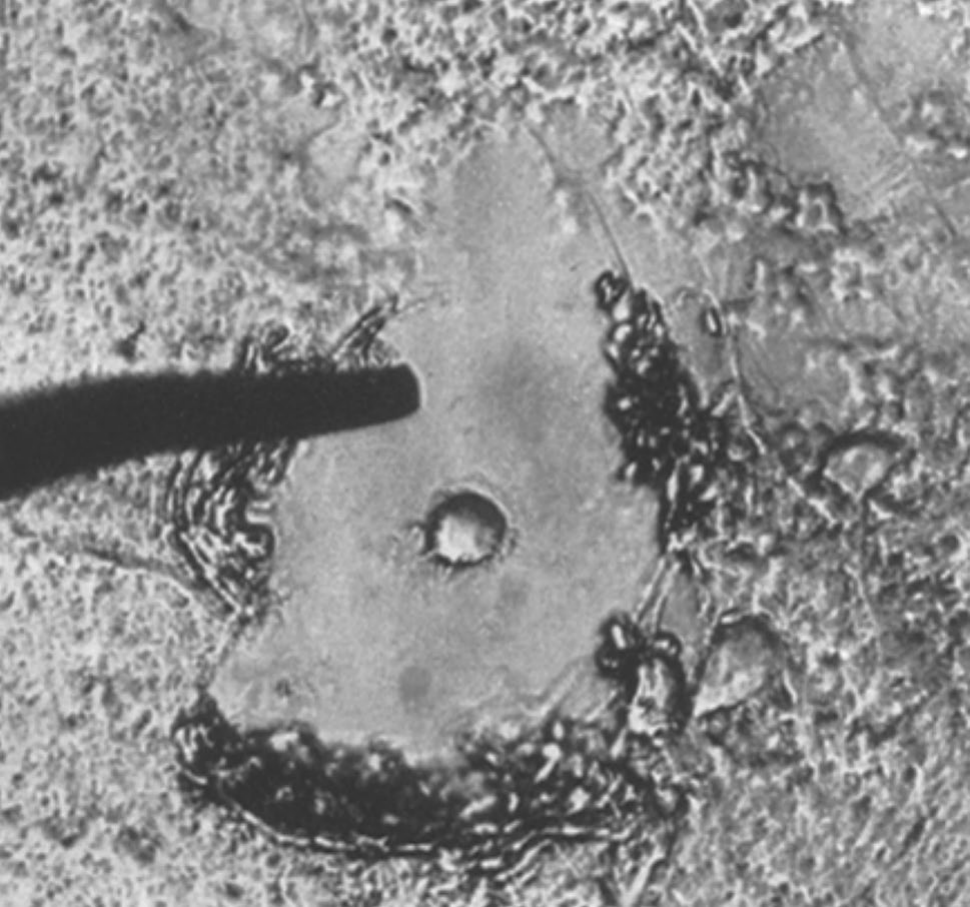
Isolation of a Purkinje cell with the “Mikromanipulator.” Figure 6 from Sandritter et al. 1966

Quantification of histochemical results to objectify the observations was in its infancy and definitely not a matter of course. Thus, the paper by Kasten (1958) to quantify the Feulgen reaction for DNA was rather exceptional at that time. A stimulative trigger for quantitative histochemistry was the thorough account of the application of the point counting method by Leibniz (1964), illustrated by the quantitative analysis of the age-related lipofuscin accumulation in neurons of the olivary body. To conclude this part of the review, we would like to refer to a paper by Sandritter and coworkers (1966) concerning aspects of tissue preparation for cytophotometric measurements of DNA, homogeneous objects, and cytoplasmic substances. Almost as an aside, the authors show one application of their special squashing technique, namely the isolation of single cells under a conventional light microscope with a micromanipulator, a pointed metal needle, for single cell analysis (Fig. 9). A decade later, laser-assisted microdissection became available for single cell analysis (Isenberg et al. 1976) and since then has become a most powerful technique in diverse types of investigations (Fujita et al. 2006; Kase et al. 2007; Sakurada et al. 2006; Walch et al. 2001).

### Towards present day histochemistry

It is certainly not an overstatement to say that quantum leaps have occurred in histochemistry since at least the 1980s, and that its focus has broadened significantly since then and that this vibrant development continues right into the present time. The basis for this enormous progress was diverse and comprised, for instance, the availability of antibodies suitable for light and electron microscopic immunolabeling, together with the introduction of new types of markers, and molecular reagents, such as genetically encoded fluorescent tags and transgenes or padlock probes, the application of mass spectrometry in histology, the development of novel types of sophisticated high-tech microscopes for intravital microscopy and for single molecule analysis, and the availability of powerful image analysis software. Several Special Issues published by *Histochemistry and Cell Biology* (Table 1), as well as the collection under the title “65th anniversary of HCB: The 50 topmost cited articles” (which is available at https://link.springer.com/collections/hddgjefcie) are testimony of these innovative developments.

**Table 1:** Special Issues published by *Histochemistry and Cell Biology*

25 Years of Colloidal Gold Labeling Editor: J. Roth, Zurich, Switzerland. Volume 106, issue 1, 1996
In Situ Hybridization and Related Techniques Editors: H. Höfler, Munich, Germany and A.K. Raap, Leiden, The Netherlands. Volume 108, issues 4 and 5, 1997
Centennial of the Golgi Apparatus Editors: Margit Pavelka, Vienna, Austria and D.J. Morré, West Lafayette, IN, USA. Volume 109, issues 5 and 6, 1998
Histochemistry in Gene Technology Editors: F.T. Bosman, Lausanne and J. Roth, Zurich, Switzerland. Volume 115, issue 1, 2001
Secretion, Endocytosis, Quality Control Editors: Margit Pavelka, Vienna, Austria and J. Roth, Zurich, Switzerland. Volume 117, issue 2, 2002
Active Oxygen and Nitrogen Species in Biology Editors: J.M. Robinson, Columbus, OH, USA, H. Seguchi, Kochi, Japan and J.A. Badwey, Boston, MA, USA. Volume 122, issue 4, 2004
Functional Structure of the Cell Nucleus Editors: P. Hozák, Prague, Czech Republic and S. Fakan, Lausanne, Switzerland. Volume 125, issues 1 and 2, 2006
In Focus: Intermediate Filaments Editors: P. Hozák, Prague, Czech Republic, P. Debbage, Innsbruck, Austria and J. Roth, Zurich, Switzerland. Volume 140, issue 1, 2013
In Focus: Golgi Apparatus Editors: Margit Pavelka, Vienna, Austria and J. Roth, Zurich, Switzerland. Volume 140, issues 3 and 4, 2013
In Focus: Single-Molecule Super-Resolution Microscopy Editors: M. Heilemann, Frankfurt a.M., Germany and J. Roth, Zurich, Switzerland. Volume 141, issue 6 and volume 142, issue 1, 2014
In Focus: The Cell Nucleus Editors: Klara Weipoltshammer and Ch. Schöfer, Vienna, Austria. Volume 145, issue 4, 2016
In Focus: From Cell Biology to Tissue Structure and Function Editor: Esther Asan, Würzburg, Germany. Volume 146, issue 6, 2016
In Focus: The Sugar Code Editors: H.-J. Gabius, Munich, Germany and J. Roth, Zurich, Switzerland. Volume 147, issue 2, 2017
In Focus: Hard Tissue Biology Editors: N. Amizuka, Sapporo and S. Kitazawa, Toon, Japan. Volume: 149, issue 4, 2018
In Focus: In Vivo Cell Biology in Zebrafish—New Insights into Vertebrate Development and Disease Editors: Steffen Scholpp and Michael Schrader, Exeter, UK. Vol. 154, issue 5, 2020
In Focus: 3D Imaging in Lung Biology Editors: Christian Mühlfeld, Hannover, Germany and Douglas J. Taatjes, Burlington, VT, USA. Vol. 155, issue 2, 2021
In Focus: Mammalian Gametogenesis Editors: Y. Hishikawa, Miyazaki and T. Takizawa, Tokyo, Japan. Volume 157, issue 3, 2022
In Focus in Vienna: Microscopy and Cellular Organelles Editors: M. Stöger-Pollach and Margit Pavelka, Vienna, Austria. Volume 158, issue 3, 2022
In Focus: Data Management and Data Analysis in Microscopy Editors: Ben N.G. Giepmans, Groningen, Katy J. Wolstencroft, Leiden, The Netherlands and D.J. Taatjes, Burlington, VT, USA. In preparation, 2023

In the following section of the review, we highlight a few papers showcasing recent progress and providing a look to the future. The examples we have chosen represent the combination of spectroscopic techniques and histology to identify, quantify, and precisely determine the tissue localization of proteins and lipids, and small molecules, such as pharmaceutical agents. For an in depth treatise on those topics, we recommend consulting the reviews by Walch et al. (2008), Pól et al. (2010), Sun and Walch (2013), Römpp and Spengler (2013), Papathomas et al. (2019), and Dawes et al. (2020).

In their original work on chemical imaging of intracellular lipid droplets in ex vivo muscle, Billecke and coworkers (2014) combined label-free, non-invasive coherent anti-Stokes Raman scattering microscopy together with multivariate, chemometric analysis. This type of spectroscopic microscopy is highly suited for studies on fresh tissue sections (Day et al. 2010) since it combines quantitative spatial information on lipid droplet size and content at submicron resolution, local chemical composition of the study material, and permits examination of interorganelle interaction. As pointed out by the authors, they were able to show an increase in lipid droplets and lipid accumulation in perilipin-overexpressing muscle of rats fed a high fat diet. The chemometric analysis of the coherent anti-Stokes Raman scattering additionally revealed mitochondrial-like features.

**Fig. 10:**
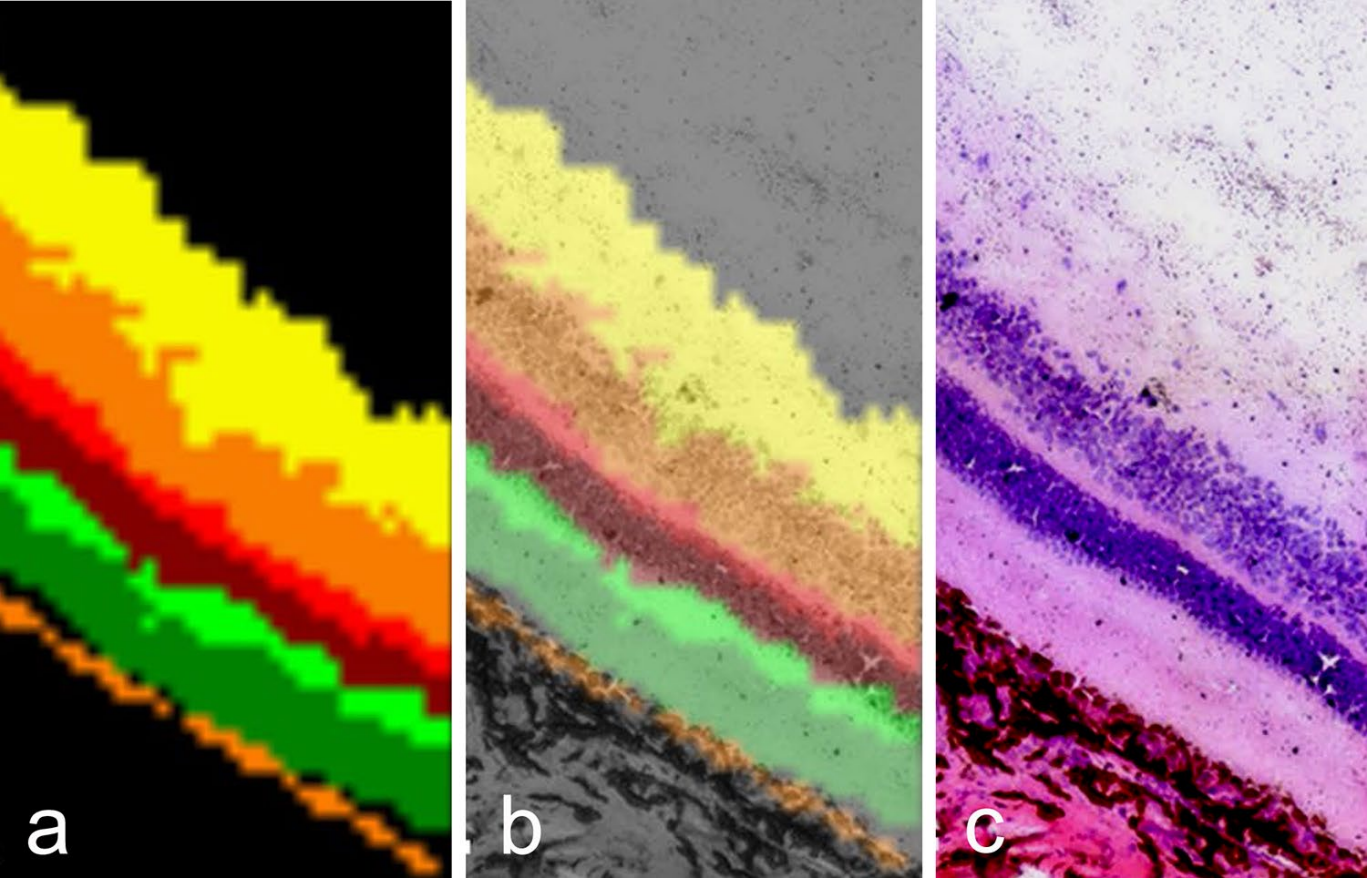
MALDI-IMS of fresh frozen porcine retina. **a** Hierarchical clustering of similar peak populations reveals distributions corresponding to different retinal layers. **b** Coregistration of hierarchically clustered masses. **c** Hematoxylin–eosin stained section. Part of Fig. 5 from Ly et al. 2015

Matrix-assisted laser desorption/ionization (MALDI) imaging mass spectrometry (IMS) represents another powerful spectroscopic technique for the label-free analysis of the in situ molecular composition of tissue sections (Caprioli et al. 1997). Ly et al. (2015) performed MALDI–IMS for the analysis of lipids in the mammalian retina, demonstrating that frozen retinal sections with sublimated matrix provided much improved spatial resolution (< 20 µm). Hence, it permitted the assignment of similar peak populations (phospholipids) by hierarchical clustering to individual retinal cell layers (Fig. 10). Similarly, MALDI imaging can also be used to detect and localize drugs in the context of the tissue environment (Balluff et al. 2011). Huber et al. (2014) successfully optimized sample preparation procedures for drug detection using an ex vivo tissue spotting approach for four drugs. The ex vivo results were validated in vivo in drug-treated mice by MALDI–TOF and MALDI–Fourier transform ion cyclotron resonance (FTICR) imaging. Therefore, the ex vivo tissue spotting method represents an efficient and reliable testing tool to economize drug analysis by MALDI imaging of tissue sections. Moreover, MALDI–FTICR imaging has been shown to be a valuable tool for label-free metabolomic studies (reviewed by Papathomas et al. 2019) as well. However, the metabolomics analysis of white adipose tissue by MALDI–FTICR, has proven difficult since the excess liquid lipids on the tissue section surface resulted in matrix disturbance and perturbed *m/z* species detection. Wang et al. (2022) were able to improve *m/z* species detection by merely removing the excess liquid lipid layer with filter paper before coating the matrix, a solution of striking simplicity. Highlighting the work of Belazi et al. (2009), we remain on the topic of adipose tissue, but pivot to time-of-flight secondary ion mass spectrometry (ToF–SIMS) as a surface analytical method (Belu et al. 2003). Here, ToF– SIMS was used for the chemical microanalysis of the binding of osmium tetroxide (OsO_4_) to lipids in mouse gluteal fat pads. Following OsO_4_ staining, two types of osmium oxide were detected on the tissue surface. Consistent with the view that OsO_4_ binds to unsaturated lipids, the OsO_3_^−^ ion colocalized with unsaturated C18 fatty acids and diglycerides containing these fatty acids. The binding to unsaturated C18 fatty acids, however, was stronger than to unsaturated C16 or C14 fatty acids. The OsO_3_(OsO)_*n*_^−^ colocalized with proteins and aliphatic phospholipid side chains. Thus, ToF–SIMS appears highly useful for disclosing the chemical background of histological stains.

We conclude this review by referring to recent work of Ritschar and coworkers (2022) combining spectroscopic techniques with machine learning to identify target tissues in the soil model organism *Eisenia fetida* for application in ecotoxicological investigations. They sequentially applied Fourier transform infrared spectroscopy (FTIR) and MALDI-IMS, followed by data analysis via random decision forest classification using random forest classifiers. The combination of FTIR and machine learning algorithms based on random decision forest classification permitted rapid chemical tissue type identification and was used to define regions of interest. MALDI-IMS was applied for high resolution lipid analysis and to assign pollutant-sensitive specific lipids to FTIR-identified different target tissues. This non-invasive multimodal analysis was proposed to provide a time-saving methodology for ecotoxicological research.

Given the recent breakneck speed in the introduction of technological innovations in microscopy imaging and analysis, as well as experimental staining protocols, we envision an exciting and bright future for *Histochemistry and Cell Biology.*

## Footnotes

1 The terminology used in the original publications is always cited.
